# Astaxanthin Delivery Across Administration Routes: Recent Advances to Improve Stability and Bioavailability

**DOI:** 10.3390/pharmaceutics18050523

**Published:** 2026-04-25

**Authors:** Laetitia Novelli, Marco Cespi, Diego Romano Perinelli, Giulia Bonacucina

**Affiliations:** Chemistry Interdisciplinary Project (ChIP), School of Pharmacy, University of Camerino, via Madonna delle Carceri, 62032 Camerino, Italy; laetitia.novelli@unicam.it (L.N.); marco.cespi@unicam.it (M.C.); giulia.bonacucina@unicam.it (G.B.)

**Keywords:** natural antioxidants, nutraceuticals, cosmetics, encapsulation, anti-aging, routes of administration, oral, topical, parenteral, chemical instability

## Abstract

Astaxanthin (ASX) is a xanthophyll carotenoid widely studied for its beneficial properties in humans, mainly related to its local or systemic antioxidant, cytoprotective and immunomodulatory effects. Particularly, ASX can donate electrons to neutralize reactive oxygen species (ROS), thereby mitigating oxidative stress, a key factor in the onset of several chronic and degenerative diseases. Thanks to these valuable properties, ASX has attracted considerable interest in the pharmaceutical, nutraceutical and cosmetic sectors. Despite its promising biological potential, the application of ASX is limited by several physicochemical factors. It is a highly lipophilic molecule, unstable when exposed to light, heat and oxygen, which leads to rapid degradation, and is characterized by low bioavailability. To overcome these limitations, various formulation strategies have been developed, particularly encapsulation-based approaches aimed at improving stability, solubility and therapeutic applications. This review provides an overview of the conventional and innovative dosage forms of ASX developed to enhance bioavailability and preserve the chemical and biological properties of this powerful antioxidant, by focusing on the different administration routes. Special attention is given to the advantages and limitations of the different formulation strategies and their implications for human health according to the different administration routes. Although oral administration remains the most explored route, further studies are needed to develop formulations suitable for alternative routes of administration.

## 1. Introduction

**Figure 1:**
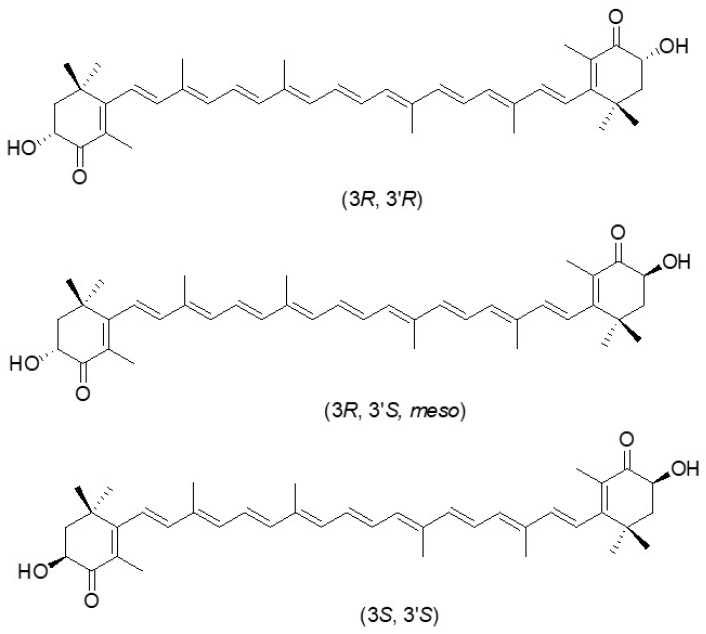
Chemical structure and stereochemistry of natural and synthetic ASX.

Astaxanthin (3,3′-dihydroxy-β,β′-carotene-4,4′-dione) is a carotenoid belonging to the xanthophyll group [1], whose chemical structure consists of a linear polyene chain characterized by conjugated double bonds and two terminal β-ionone rings, each bearing a keto (C=O) and a hydroxyl (–OH) functional group (Figure 1) [2]. Owing to its chemical structure, astaxanthin (ASX) can exist in different isomeric forms: in nature, the predominant geometric isomer is the all-trans form (all-E-ASX) [3], while the most common stereoisomer is the 3S,3′S configuration, determined by the presence of two stereogenic carbon atoms at the C3 and C3′ positions [4]. ASX occurs in different molecular forms: besides the free form, with unmodified hydroxyl groups, it can be bound to proteins and lipoproteins in animals, or present as glycosides in bacteria [5,6]. This unique chemical structure strongly influences the biological properties of ASX. The conjugated double bonds confer exceptionally high antioxidant activity, enabling the molecule to donate electrons and neutralize reactive oxygen species (ROS) [5]. Several studies have shown that the antioxidant capacity of ASX can be up to 6000 times greater than that of vitamin C [7], and it also surpasses that of β-carotene and α-tocopherol [8]. Thanks to this potent antioxidant action, ASX helps protect cells from oxidative damage, modulate inflammatory processes, strengthen immune defenses, promote DNA repair, slow cellular aging, and support the health of the cardiovascular system, liver, eyes, and skin [9].

This wide range of biological activities makes ASX a molecule of considerable interest. While its pharmaceutical applications are still limited and mostly at the research stage, it is already employed in the nutraceutical and cosmetic fields [10]. Its broader use, however, is constrained by several chemical and physical limitations. ASX is a highly hydrophobic molecule and therefore poorly soluble in polar solvents, including water. This poor solubility hinders its dissolution in gastrointestinal fluids and limits its absorption via the oral route [11]. In addition, its polyene structure, rich in unsaturated conjugated double bonds, makes the molecule particularly unstable [12] and highly susceptible to degradation by oxidation, light, heat, and acidic pH [3], which can also compromise its stability in topical formulations. These limitations, low bioavailability and susceptibility to degradation, compromise the effectiveness of ASX’s biological activities. To preserve its integrity and improve its apparent solubility in aqueous media, appropriate formulation strategies are therefore necessary. Conventional formulations, such as oil suspensions and softgel capsules, could enhance the chemical stability of ASX by forming a protective barrier against adverse environmental factors, but they are unable to improve bioavailability [13]. In contrast, innovative delivery systems, including formulations based on nano- and micro-carriers, appear to significantly enhance both stability and oral absorption, thereby improving the effectiveness of ASX [14].

Due to the issues arising from ASX’s low bioavailability and chemical instability, several reviews in the literature focus on the different delivery systems developed for ASX, distinguishing conventional approaches from innovative ones. They specifically considered the molecule’s chemical structure, its beneficial properties for humans, and the critical issues related to its sensitivity to oxidation, light, and heat, for which the development of suitable encapsulation approaches is required [15,16,17,18]. Differently, the purpose of the present review is to summarize the available delivery strategies for ASX, both conventional and innovative ones, by focusing on the different routes of administration and discussing their potential to maintain ASX’s biological and chemical properties, highlighting implications for human health.

## 2. Astaxanthin Chemical–Physical Properties, Sources and Extraction

ASX has a highly lipophilic structure due to its conjugated polyene chain and terminal low polar ionone rings with a molecular weight (MW) of 596.84 g/mol. It exhibits very low aqueous solubility (close to or below µg/mL) at 25 °C, reflecting its strong hydrophobicity, and its solubility under acidic conditions is 10–20 times higher than under neutral and basic conditions [19]. On the other side, it has higher solubility in organic solvents, and it is generally miscible in lipid matrix. Its solubility in organic solvents decreases in the order of dichloromethane > chloroform > dimethyl sulfoxide > acetone > methanol > ethanol. Moreover, ASX has a high partition coefficient (logP) with reported (calculated) logP values typically ranging from approximately 8 to 10, depending on the prediction method used, and even higher theoretical estimated values (logP ≈ 12–13) reported in the literature [20]. ASX remains mainly non-ionized across a wide range of pH values (including physiological one), due to the lack of strongly ionizable functional groups; therefore, a reliable experimental pKa is not reported in the literature, but only predicted values, with approximate pKa of ≈12–13 [21]. As ASX is largely non-ionized, the distribution coefficient (logD) is expected to be similar to logP (logP ≈ logD).

ASX is naturally found in a variety of organisms, including microalgae, yeast, crustaceans, fish, and some insects, where it contributes to pigmentation and offers antioxidant protection. Among these, the primary natural source of ASX for human use is *Haematococcus pluvialis* (class Chlorophyceae), a green, unicellular, freshwater microalga [22]. Under environmental stress conditions, such as nitrogen and phosphorus limitation, high temperature, high salinity, and strong light exposure [23], *H. pluvialis* accumulates high amounts of ASX in its red cysts [24]. To date, only the natural ASX produced by *H. pluvialis* has received Food and Drug Administration (FDA) approval for human consumption [25]. Other natural sources of ASX include the yeast *Phaffia rhodozyma*, which produces ASX through controlled fermentation [25,26]; certain bacteria, such as *Paracoccus carotinifaciens* [20]; and by-products from crustacean processing, such as shrimp and crab, from which ASX can be recovered from edible parts [26,27]. However, these natural alternative sources have limitations for commercial human use, including low production yields and the complexity and cost of the extraction and production processes [21]. Additionally, natural ASX from krill, *Phaffia rhodozyma* yeast or *Paracoccus* bacteria are mainly used in non-human applications such as aquaculture, poultry and livestock farming, where less purified and more economically sustainable products are sufficient [28].

ASX obtained from natural sources requires a subsequent extraction process to ensure its recovery without compromising quality. Over time, several extraction methods have been evaluated. Traditionally, ASX is recovered by solid–liquid extraction using organic solvents such as acetone, ethanol, methanol, ethyl acetate, dichloromethane and hexane. Among these, polar solvents like ethanol exhibit good cell membrane penetration and high solubility for ASX [29]. ASX ethanolic extracts facilitate the preparation of homogeneous formulations and enhance the bioavailability of the ASX in the final product. Nevertheless, the use of polar solvents may also present some limitations, as they are not always compatible with certain formulations, particularly those with a lipid or oily base [30]. It has been observed that solvent extraction becomes more efficient when the biomass is pretreated with acid, as this step helps disrupt the cell walls and facilitates the release of ASX. In particular, when *H. pluvialis* biomass was treated with HCl prior to extraction with acetone, a yield higher than 85%(*w*/*w*) was achieved. However, at higher acid concentrations (>4 N HCl), the ASX content significantly decreased, probably due to acid-induced degradation of the compound or reduced solubility in highly acidic environments [31]. A similar approach was applied also to the yeast *Phaffia rhodozyma*, using different acid pretreatments (hydrochloric, lactic, or acetic acid). Among these, lactic acid proved most effective, promoting efficient cell disruption while minimizing ASX degradation [32]. Another extraction technique is the use of supercritical CO_2_ (scCO_2_), which is increasingly adopted at industrial scale as an efficient and environmentally friendly method for recovering ASX. This technique is appreciated for being relatively inexpensive, non-toxic, chemically inert, and stable compared to other solvents [33]. The absence of organic solvents results in ASX with a high degree of purity, suitable for cosmetic, nutraceutical, or pharmaceutical formulations. Moreover, scCO_2_ extraction minimizes thermal and oxidative degradation of ASX, improving the safety of the final product. However, the extract obtained with CO_2_ is usually poorly soluble in aqueous or polar formulations, requiring the use of additional excipients or specific delivery systems to enhance bioavailability [34]. When CO_2_ is used alone, its low polarity limits the extraction efficiency of xanthophylls such as ASX, since the gas preferentially dissolves non-polar compounds [35]. The addition of small amounts of polar co-solvents, such as ethanol or methanol, increases the overall polarity of the supercritical fluid and significantly enhances both the rate and yield of ASX extraction [12,36]. Additionally, it seems that enzymatic pretreatment can also boost scCO_2_ extraction, improving the recovery of ASX and making the process more efficient from a formulation perspective [36]. More recently, CO_2_-based ionic liquids (ILs) have been proposed to enhance scCO_2_ extraction. These ILs permeabilize the robust cell walls of *H. pluvialis*, facilitating astaxanthin recovery while being distillable for easy recycling. This approach combines the advantages of ILs with CO_2_ extraction, offering an efficient and sustainable method for obtaining ASX from microalgal biomass [37].

Further innovations involve the use of ultrathin α-quartz nanoplates (NPLs) as selective adsorption surfaces for ASX. These nanostructures can induce microlesions in the cell wall, facilitating compound release, while specific solvents or mild physical treatments such as light, heat, or sonication are applied for subsequent extraction. When combined with conventional solvents such as DCM/methanol, this approach has achieved nearly complete ASX recovery [38]. The synergistic use of CO_2_-based ionic liquids and α-quartz NPLs allows extraction at room temperature, minimizing thermal degradation and enhancing environmental sustainability. However, the ILs must still be removed from the final extract, typically using organic solvents, introducing a potential risk of contamination [39]. The selection of an appropriate extraction method is crucial for obtaining ASX products that combine high biological activity with optimal stability and bioavailability. Advances in extraction technologies, from solvent-based and scCO_2_ methods to ionic liquids and α-quartz nanoplates, demonstrate how the choice of technique can strongly influence both the quality and safety of the final formulation.

In addition to natural sources, astaxanthin can also be produced through chemical synthesis. Synthetic ASX is widely used in aquaculture and animal feed due to its lower production costs and high availability. However, it differs from natural ASX in terms of stereoisomer composition and esterification, which can affect its bioactivity and functional properties [40]. Specifically, synthetic ASX consists of a 1:2:1 mixture of isomers (3S, 3S), (3R, 3S), and (3R, 3R) respectively, with a markedly lower antioxidant capacity (14–55 times weaker) than natural ASX composed selectively of (3S, 3′S)-isomer (from the microalgae *H. pluvialis*) or (3R, 3′R)-isomer (from the yeast *Xanthophyllomyces dendrorhous*). Moreover, natural ASX is composed of ~95% mono-esterified and di-esterified molecules, while synthetic ASX predominantly exists in a non-esterified form [3].

Due to the different chemical structure, lower bioactivity and lack of long-term safety and efficacy studies on humans, synthetic ASX has not yet been approved as a human food supplement in the EU, nor has it received GRAS status in the USA [2], and that from *H. pluvialis* remains the only version usable for human consumption [41].

## 3. Biological Activities

ASX, thanks to its potent antioxidant properties and low toxicity, is used to prevent or treat degenerative, inflammatory, and traumatic diseases. It is also widely used in cosmetic applications aimed at protecting the skin and promoting its overall health. The antioxidant activity of ASX comes from a complex mechanism that combines direct scavenging and quenching effects with the modulation of antioxidant signaling pathways involving endogenous enzymes and the promotion of mitochondrial function protection. ASX protects mitochondria from high levels of reactive oxygen species (ROS) and preserves its integrity by maintaining membrane potential and limiting the opening of the mitochondrial permeability transition pore (mPTP). ASX also exerts an interesting effect in cell apoptosis, showing a dual role: it prevents apoptosis in normal or stressed healthy cells while triggering it in cancer cells [42]. Overall, ASX’s main actions in the human body include counteracting the formation of ROS, the molecules responsible for oxidative stress, and enhancing the endogenous defense systems through regulating the expression of enzymes involved in the cellular stress response, such as heme oxygenase-1 (HO-1). These activities contribute to the potentiation of the cellular and systemic antioxidant defense strategies [43]. All these properties make ASX a promising candidate for the prevention and treatment of various conditions associated with aging and oxidative damage such as chronic inflammation and neurodegenerative or cardiovascular diseases as well as metabolic disorders and diabetes. In addition to the antioxidant activity, ASX supports immune function and has been shown to reduce the symptoms of *Helicobacter pylori*-induced ulcers by alleviating gastric inflammation [9]. ASX exerts protective effects on the visual system, safeguarding retinal photoreceptors from degeneration and counteracting ischemic damage caused by high intraocular pressure. If left untreated, such damage can lead to serious conditions, including diabetic retinopathy, cystoid macular edema, central retinal arterial or venous occlusions, and glaucoma. Experimental studies in rats demonstrated that ASX can cross the blood–retina–brain barrier, despite not being naturally present in the retina. Its accumulation in retinal tissue was associated with reduced phototoxic damage, as evidenced by increased levels of rhodopsin, the photosensitive protein essential for vision [44]. Additionally, ASX helps slow the progression of degenerative eye diseases, such as age-related macular degeneration, and supports the maintenance of visual function [45]. At the neurological level, dietary ASX supplementation may help protect the brain by reducing ischemic damage through the inhibition of oxidative stress, limiting glutamate release, and preventing apoptosis. Recent studies suggest that ASX could be particularly effective in the early stages of subarachnoid hemorrhage and may have potential in the treatment of neurodegenerative diseases, such as Alzheimer’s disease, by inhibiting the aggregation of amyloid β peptide (Aβ), a key factor in the disease’s pathogenesis [45,46]. In the liver, ASX has demonstrated significant protective and therapeutic effects against a range of disorders, including liver damage, fibrosis, and liver cancer [47]. For example, Chen et al. (2015) reported that supplementation with free-form ASX powder (FFAP) in a high-cholesterol diet enhanced liver antioxidant activity, increasing hepatic catalase activity as well as levels of vitamin C, vitamin E, and glutathione (GSH) [48]. In vitro studies on the HepG2 cell line showed that ASX derived from *H. pluvialis* can inhibit cancer cell proliferation, induce DNA damage, and trigger apoptosis [49]. Additionally, ASX positively modulates the blood lipid profile by raising HDL cholesterol while reducing triglycerides, LDL cholesterol, and apolipoprotein B [45]. ASX also supports immune function. In rats, its administration enhanced the cytotoxic activity of natural killer (NK) cells, which are crucial for immunosurveillance against tumors and virus-infected cells. This immunomodulatory effect may contribute to protection against certain cancers, including breast, colon, and urinary tract malignancies [9]. Finally, ASX exerts beneficial effects on the skin. During aging, oxidative stress caused by ROS compromises skin structure by damaging DNA, triggering inflammatory responses, and stimulating the production of matrix metalloproteinases (MMPs), enzymes that degrade collagen and elastin in the dermis [38,43]. Both oral supplementation and topical application of ASX have been shown to improve skin appearance, reducing wrinkles and age spots, increasing elasticity and hydration, and promoting better regulation of sebum production [50].

Taken together, the extraordinary properties of ASX and its wide-ranging benefits for human health highlight the need to develop pharmaceutical, nutraceutical, and cosmetic products that can overcome challenges related to the molecule’s instability and bioavailability, while ensuring efficacy and safety in the final formulations.

## 4. Routes of Administration of ASX and Related Dosage Forms

ASX is a natural compound that finds applications in different fields such as pharmaceutics, nutraceuticals and cosmetics in relation to human health and wellness. According to the use, it can be administered through a variety of dosage forms, including capsules, softgels, tablets, powders, creams, water- or oil-based liquids, and extracts. Each formulation is designed to ensure that the active ingredient reaches its target site effectively and safely. To do this, encapsulation is the most common strategy to improve the chemical stability of the molecule, which is particularly sensitive to heat, oxygen and light exposure, leading to oxidative, photochemical, and thermal degradation. The mechanisms underlying ASX protection through encapsulation include physical shielding from oxygen and light, reduction of molecular mobility, and stabilization through lipid or polymeric interactions, which collectively enhance the chemical stability and functional performance of ASX [51].

The topic regarding the encapsulation of ASX has been largely reviewed [52,53,54,55]. However, the route of administration also plays a crucial role in determining the absorption, distribution, and overall bioavailability of ASX [56,57]. Factors such as the desired onset and duration of action, the characteristics of the target tissue, and the intended health or cosmetic benefit all influence this choice [58]. Consequently, the selection of the administration route and the corresponding dosage form is a key step in optimizing the efficacy of ASX-based products in relation to their specific applications. These aspects will be examined in detail in the following sections, by discussing only the available studies referring to delivery strategies of ASX related to specific routes of administration.

### 4.1. Oral Administration

**Figure 2:**
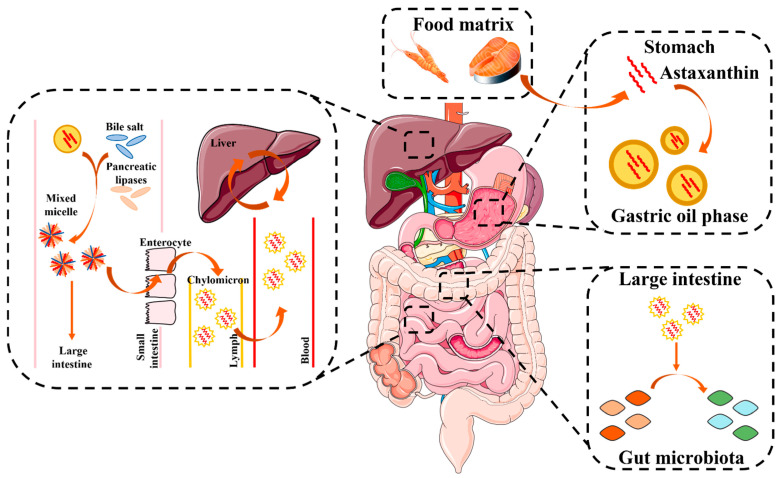
Absorption, transport and metabolism of ASX. Reproduced from [65].

Oral administration is the most common and convenient route for delivering bioactive compounds such as ASX, both for pharmaceutical and nutraceutical purposes, due to its non-invasive nature and high consumer acceptance [59]. After ingestion, ASX is absorbed primarily in the small intestine, where the large surface area and lipid-rich environment favor the uptake of lipophilic molecules [60,61]. In this way, this powerful antioxidant passes through the gastrointestinal tract and enters in the systemic circulation, distributing throughout the body until it reaches internal organs such as the liver, kidney, pancreas, lung and prostate, as shown in Figure 2 [62]. This enables widespread antioxidant and anti-inflammatory effects that cannot be achieved through topical application, which acts exclusively at the surface. The topic of oral delivery of ASX has been recently reviewed [63,64].

In this context, solid oral dosage forms offer additional advantages since they are easy to take, have enhance stability and assure accurate and uniform dosing. However, the oral bioavailability of free ASX is low, due to its poor aqueous solubility and chemical instability, and it can be influenced by several physiological factors, such as gastric acidity, digestive enzymes, bile salts and the presence of intestinal mucus, all of which can reduce its stability and absorption [66]. As a result, the amount of ASX that reaches systemic circulation can vary depending on the formulation and dietary conditions. In particular, the co-administration with lipid-based formulations, likely simulating the effect of lipid-rich foods, significantly enhances its absorption and overall bioavailability. Specifically, all the three tested lipid-based formulations [long-chain triglyceride and polysorbate 80 (formulation A), glycerol mono- and dioleate and polysorbate 80 (formulation B), and glycerol mono- and dioleate, polysorbate 80 and sorbitan monooleate (formulation C)] all showed an improved bioavailability that is 1.7 to 3.7 times higher than the reference formulation (powder in hard capsules) [67].

Moreover, the development of formulations such as lipid-based carriers, emulsions and encapsulated systems can protect ASX from degradation and further improve its bioavailability and bioaccessibility [52]. Indeed, the encapsulation of carotenoids is an effective strategy to improve their physicochemical properties, including water solubility, stability during storage, controlled and sustained release, bioaccessibility, bioavailability and bioactivity [68]. Among the various dosage forms, capsules represent a particularly advantageous administration method for ASX, allowing the delivery of a compound characterized by poor water solubility and limited intestinal absorption [69]. However, to maximize absorption efficiency and ensure consistent bioavailability, further formulation optimization is required. Conventional solid dosage forms are generally cost-effective and widely accepted, but their efficacy is influenced by multiple factors, including the formulation technologies employed, the characteristics of the active ingredient and the properties of the capsule shell. These variables affect the release profile and absorption of the compound [70] and can be further modulated by physiological conditions within the gastrointestinal tract, particularly pH, which impacts the overall performance of the delivery system [71]. ASX is a fat-soluble compound that follows the same absorption pathways as dietary lipids. Consequently, its bioavailability increases in the presence of fats and is higher when administered in lipid- or oil-based formulations, such as softgels, compared to hard capsules [67], while the stability of ASX formulated in capsules is highly dependent on the composition and type of formulation. When ASX is incorporated as powder into solid formulations, it is typically administered in hard capsules. These systems are generally suitable for dry formulations and may exhibit good physical stability under controlled low-moisture conditions. However, the assumption that hard capsules can inherently provide higher stability than soft gelatin capsules is an oversimplification, as stability depends on both environmental factors and formulation characteristics. While hard capsule shells are less hygroscopic and could show lower chemical reactivity, they can be susceptible to brittleness under low humidity and do not necessarily provide superior protection against oxidative degradation of the active compound. Conversely, soft gelatin capsules containing solid fills can also present stability challenges due to potential interactions between the fill material and the shell, as reported by Palomero-Hernández [72]. Despite these limitations, when ASX is formulated in liquid form, typically dispersed in oily matrices, softgels are often considered the most appropriate dosage form. Their hermetically sealed structure can limit oxygen exposure and enhance the chemical stability of lipophilic and oxidation-sensitive compounds such as ASX, thereby improving overall stability under these conditions (Table 1) [73]. Supporting this evidence, a study conducted in healthy male volunteers [67] demonstrated that intake of an ASX supplement after breakfast significantly increased its bioavailability. Under these conditions, bile salt secretion and pancreatic lipase activity, stimulated by the presence of food and lipids in the gastrointestinal tract, were optimized. Furthermore, the addition of oils and/or surfactants to the formulation contributed to a further improvement in this parameter. Given that gastrointestinal absorption of ASX is generally low [74], the enhancement observed following a high-fat meal highlights the importance of lipid-based formulations [75]. The release profile of ASX is better in softgels than in hard capsules. In this context, Kanakaraj et al. (2024) developed softgel formulations capable of achieving a release rate of 96.8%, with recovery values ranging from 97.7% to 100.1%, demonstrating excellent dissolution performance [76].

In addition to conventional softgels containing lipid-based formulations, more complex encapsulation systems have recently been explored, which have been extensively reviewed [77]. Wuytens et al. (2014) designed multilayer polyelectrolyte capsules capable of incorporating multiple active ingredients and modulating their release through a polymeric membrane [71]. Similarly, Zhao et al. (2022) proposed a protein–polysaccharide complex-based delivery system, using bovine serum albumin and chitosan as carriers for ASX aggregates [78]. These nanoparticles showed encapsulation efficiencies exceeding 90%, representing a promising approach for the development of water-dispersible ASX formulations. Zhu et al. developed a novel nanocarrier based on polyethylene glycol-grafted chitosan (PEG-g-CS) to enhance the intestinal bioavailability of astaxanthin ~6 times in comparison to free ASX [79]. In another work, ASX-loaded composite micelles were successfully prepared via coaxial electrospray technology and demonstrated an increase in the intestinal bioavailability of around 5 times [80]. Sorasitthiyanukarn et al. fabricated chitosan oligosaccharide/alginate nanoparticles loaded with ASX using oil-in-water emulsification followed by ionotropic gelation, showing a better storage stability at 4 °C for 60 days. The release rate of ASX in nanoparticles was higher (63% in 36 h) under a lower pH value (pH 1.2) compared with the neutral or alkaline medium during the in vitro release study [81]. Carboxymethyl chitosan-based nanoparticles loaded with ASX (CMC-AXT-NPs) were developed for the oral treatment of ulcerative colitis. In vitro studies showed that CMC-AXT-NPs have antioxidant and anti-inflammatory activities. In vivo studies further confirmed that CMC-AXT-NPs can reduce the clinical symptoms of colitis and suppress the secretion of pro-inflammatory cytokines [82].

Overall, few studies are currently available on the stability, biochemical behavior, bioavailability, pharmacokinetics and toxicology of ASX capsules, suggesting that both their long-term health effects and optimal dosing strategies are not yet fully established [83]. The formulation of ASX in its raw form into solid pharmaceutical dosage forms, such as tablets, presents numerous challenges. These include poor powder flowability and low apparent density, which compromise direct compression and hinder the production of homogeneous tablets, as well as issues related to the chemical stability of ASX, which is susceptible to degradation processes. Furthermore, its limited solubility in aqueous media represents an additional limiting factor, as it negatively affects gastrointestinal absorption of the compound (Table 1) [54].

Although studies on tablet formulations containing ASX are still limited, several investigations have focused on addressing the main challenges associated with this molecule, particularly by improving the critical properties required for effective formulations. To enhance ASX stability and bioavailability, it is often first subjected to microencapsulation processes or formulated as self-microemulsifying drug delivery systems (SMEDDS) prior to compression. This system, once dispersed in the gastrointestinal tract, spontaneously generates oil-in-water microemulsions in which the drug is solubilized [84]. For example, Li et al. (2024) improved the solubility, dissolution rate and oral bioavailability of ASX by formulating it as amorphous solid dispersions (ASDs) using specific excipients and surfactants, such as OSA–starch (octenyl succinic anhydride modified starch), HPMCAS-HF (hypromellose acetate succinate), Soluplus^®^, and Span 20 [85]. The association of ASX with these excipients inhibited its recrystallization into the crystalline form, which is characterized by lower solubility. Furthermore, the presence of hydrogen bonding and hydrophobic interactions between ASX and the polymeric matrix favored the formation of homogeneous ASD preparations, making these formulations promising for the delivery of hydrophobic bioactive compounds. The development of ASD had a protective effect on ASX and improved its physical and chemical stability by decreasing its Gibbs free energy through interactions with excipients at an optimized ratio. The freshly prepared ASX ASDs and ASX bulk powder were sealed in a brown vial and stored with a relative humidity of 60% at 25 °C in darkness for 30 days to evaluate the stability. Building on the concept of microencapsulation, a recent study investigated ASX encapsulated in microcapsules and subsequently formulated it into effervescent tablets via wet granulation and compression. This approach enhanced the chemical stability of ASX and the resulting tablets exhibited excellent dissolution performance, with over 90% of the active ingredient released within 2 h, while cytotoxicity tests confirmed their good biocompatibility [86]. Similarly, Aung et al. (2022) demonstrated that tablets incorporating ASX-loaded SMEDDS significantly improved the dissolution profile, achieving a release rate exceeding 98% [87]. These formulations also enhanced antioxidant activity, absorption and cellular uptake in intestinal Caco-2 cells. Furthermore, the tablets maintained both chemical and physical stability over a three-month period, highlighting SMEDDS as a promising strategy for delivering highly lipophilic compounds while improving solubility, permeability and bioavailability. A stability study of ASX SMEDDS tablets was performed under two different conditions (30 ± 2 °C/RH 65 ± 5% and 40 ± 2 °C/RH 75 ± 5%) for a different storage time up to three months.

In addition to conventional SMEDDS-based tablets, orodispersible tablets (ODTs) offer an innovative dosage form that addresses swallowing difficulties. ASX-containing ODTs have been formulated using diluents such as F-Melt^®^ Type C and F-Melt^®^ Type M. The resulting tablets displayed rapid disintegration and dissolution, with over 80% of the active ingredient released within 2 min, as well as low friability even at doses of 20–40 mg of ASX. These findings support the potential of ASX-based ODTs as viable pharmaceutical or nutraceutical products [88]. Overall, ASX encapsulation represents an effective strategy to overcome the limitations associated with its instability and poor bioavailability [89]; however, the extent of improvement and the mechanism of action depend on the structural nature of the encapsulation system. In particular, liposomal systems improve the long-term stability and aqueous solubility of ASX, preventing its degradation during administration [90]. Polymeric and lipid systems allow controlled release over time, associated with good dispersibility, stability and high cytocompatibility [91,92], while nanostructured lipid formulations (SLN) promote increased oral bioavailability due to improved stability during gastrointestinal transit. The increased stability can be attributed to the incorporation of ASX into the lipid matrix via recrystallization of stearic acid during the refrigeration process. Furthermore, ASX tends to be inactivated by isomerization under acidic and alkaline conditions, but SLNs provide effective protection against this process. Consequently, improved chemical, thermal and oxidative stability is observed, the latter being evaluated in an aqueous H_2_O_2_ solution [93]. Among lipid nanosystems, nanostructured lipid carriers (NLCs) are particularly promising, as they effectively protect ASX from external factors such as oxygen, heat and ultraviolet radiation [94], thereby contributing to the preservation of its antioxidant activity [95]. Overall, the available evidence confirms that the choice of encapsulation system is a determining factor in optimizing the performance of ASX and supports its use in oral formulations.

### 4.2. Topical Administration

**Figure 3:**
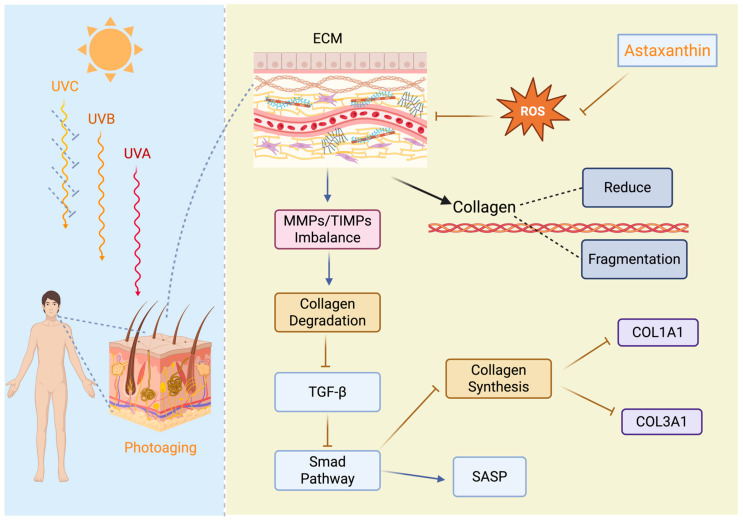
Molecular pathways underlying the stimulation of collagen production as anti-photoaging effect exerted by ASX. Reprinted from [106].

Topical administration is a widely used therapeutic modality in clinical practice, as it produces a localized effect on specific areas of the body such as the skin, eyes, nose, throat and vagina. Because the action of the active ingredient is limited to the application site and does not enter the systemic circulation, this route of administration avoids first-pass hepatic metabolism and reduces the risk of systemic side effects and gastrointestinal irritation [96]. For these reasons, topical therapy is often the preferred choice for the treatment of many clinical conditions. Although topical administration is generally associated with short-lived local drug levels, prolonged exposure can be achieved through repeated applications or continuous perfusion [97,98]. Furthermore, this non-invasive therapeutic modality stands out for its simplicity, convenience and suitability for self-administration, making it particularly appropriate for the management and treatment of various pathologies [99]. In this context, ASX can be used as an ingredient in topical products such as creams, gels, sprays and eye drops, owing to its well-recognized biological properties, as recently reviewed [100,101,102,103]. At the skin level, ASX is widely used in cosmetics and dermatology for its antioxidant, anti-inflammatory, and photoprotective properties (Figure 3). It has also been shown to be effective against premature aging induced by oxidative stress, making it a promising compound for the prevention of wrinkles and hyperpigmented spots associated with intrinsic skin aging [104,105].

**Figure 4:**
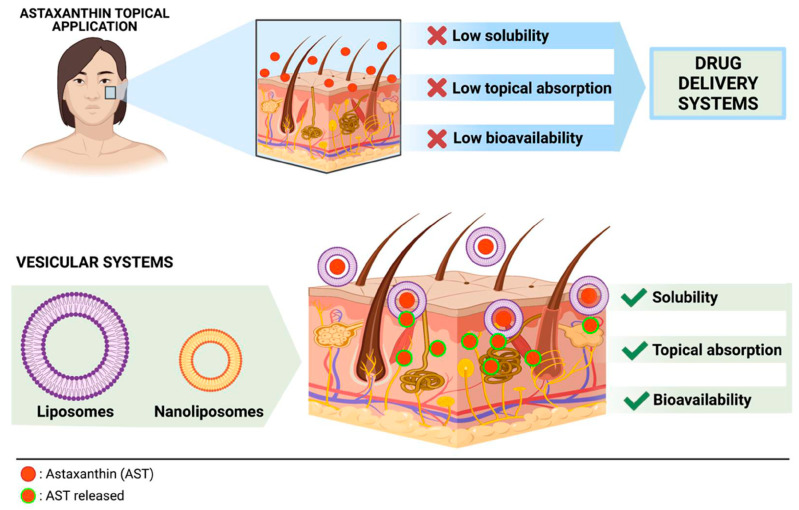
Schematic illustration of ASX skin permeation and the use of formulation strategies to enhance its skin absorption. Reprinted from [100].

At the pharmacological level, ASX has demonstrated therapeutic effects in preclinical models of atopic dermatitis, reducing the release of pro-inflammatory cytokines associated with the disease [107]. In another study, a concentration of 1 mg/mL of ASX promoted skin wound healing by enhancing collagen synthesis in human dermal fibroblasts and inhibiting the expression of matrix metalloproteinases MMP-1 and MMP-3, enzymes involved in collagen degradation [108]. A more recent study developed Carbopol-based gel containing ASX loaded into chitosan-coated nanostructured lipid carriers (CS-NLCs) intended for wound healing and tissue regeneration. This innovative system demonstrated excellent stability, biocompatibility, sustained antioxidant properties, and improved skin permeability. The encapsulation efficiency was greater than 85.45%, with a loading content of 4.04% for AST-NLCs, offering a promising formulation capable of overcoming the limitations associated with free ASX. The in vivo studies demonstrated accelerated wound healing and tissue regeneration, thanks to enhanced collagen deposition, more effective neovascularization, faster epithelialization, and reduced inflammatory cell infiltration compared to the control group [109]. Excessive UV exposure is known to induce skin damage through increased production of ROS, melanocyte proliferation, sunburn, dryness and elastin fragmentation [110]. Thanks to its high UV absorption capacity and strong antioxidant activity, ASX is considered a valuable booster agent capable of improving the effectiveness of traditional sunscreen products. Formulation studies have shown that incorporating ASX into sunscreens increases the SPF value without significant alterations in pH or viscosity, while maintaining good stability of the formulation at room temperature (25 °C) [111]. In the study by Tosato et al. (2016) [112], ASX was incorporated into phospholipid liposomes (ASX-lipo) and applied topically before UV exposure. The results showed a reduction in UV-induced skin thickening and inhibition of collagen loss, leading to visible improvement in skin appearance. Furthermore, topical applications of ASX-lipo reduced levels of malondialdehyde (MDA) and interleukin-6 (IL-6), key mediators of oxidative damage and inflammation induced by acute UVB exposure [110]. In cream formulations containing ASX, ingredients such as emulsifying wax, glycerin, stearic acid and sodium benzoate have been used to improve stability and consistency. In these formulations, ASX was shown to reduce ROS levels and tyrosinase activity, a key enzyme in melanin synthesis, thereby contributing to the reduction of hyperpigmentation [113]. However, the use of ASX presents challenges related to its stability, as discussed in the previous chapter. In this context, self-nano-emulsifying drug delivery systems (SNEDDS) or the incorporation of ASX in liposomal lipid bilayers represent a promising strategy to enhance the skin penetration of the highly lipophilic ASX into the epidermis and dermis, where it can exert its therapeutic activity (Figure 4). A SNEDDS-based formulation has been successfully prepared to promote the skin penetration of ASX and enhance its antioxidant and anti-inflammatory activity. The negative zeta potential of these formulations increases electrostatic repulsion between droplets, thereby enhancing overall physical stability. The stability of ASX SNEDDS was determined over 4 weeks of storage at room temperature when exposed to or protected from light. Physical appearance and the ASX content were determined after storage at 22–25 °C in clear vials and protected from light in amber vials. There was no sedimentation or phase separation in any SNEDDS formulation when observed immediately after formulation or after up to 28 days storage at room temperature. ASX% remaining after 30 days was in the range of 86.67–93.33% for both dark and light conditions for the SNEDDS formulations [102].

Finally, ASX has also been used in decorative cosmetics, for example as a natural colorant in cream blush formulations [114], or in facial masks containing 20 ppm ASX and characterized by a half-life of 70.7 weeks. These masks maintain a pH between 5 and 8, consistent with the slightly acidic pH of human facial skin. ASX has also been incorporated into lipsticks with demonstrated antioxidant, anti-inflammatory, anti-wrinkle and photoprotective properties [115]. In a clinical study [42], the combination of oral supplementation (6 mg/day) and topical application (2 mL/day of a 78.9 μM solution) significantly improved skin condition across all skin layers in both male and female subjects.

**Figure 5:**
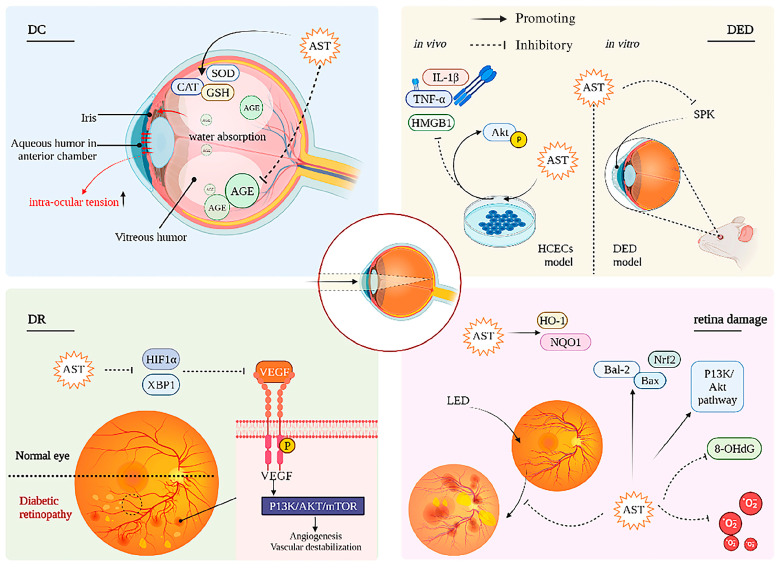
The mechanisms of ASX eye protection. ASX may protect the eye through several mechanisms including PI3K/Am. Reproduced from [118].

ASX is also effective in the prevention and treatment of several ocular diseases, including retinal diseases, ocular surface disorders, uveitis, cataracts and asthenopia [116]. Drugs administered topically as eye drops or ophthalmic ointments offer several advantages, including minimal invasiveness and good patient acceptability. However, this route of administration is limited by the rapid elimination of the drug from the ocular surface due to reduced tear volume and continuous tear drainage, as well as by the poor solubility of ASX in aqueous media [117]. In particular, topical application of ASX has been shown to limit UV radiation-induced ocular damage (Figure 5).

Experimental studies have demonstrated that the number of apoptotic cells is significantly lower in UV-irradiated corneas treated with ASX-containing eye drops compared to controls, suggesting that topical administration may provide more effective protection of the ocular surface against UV radiation than systemic administration. Currently available preclinical and clinical evidence supports the potential use of ASX in the prevention and treatment of the above-mentioned ocular diseases; however, to maximize its therapeutic efficacy, further long-term studies are required to define the optimal dosage, the most appropriate route of administration and the final composition of ophthalmic formulations [116]. To date, studies evaluating the topical ophthalmic formulations of ASX remain limited. Among these, one study described the encapsulation of ASX in a liposomal system using rabbit corneal tissue as an experimental model. The results demonstrated a significant improvement in corneal permeation compared to free ASX, along with increased release and diffusion of the active ingredient across the corneal barrier. Overall, these findings indicate that liposomal systems may represent an effective strategy for ocular delivery of ASX [119]. Additional studies have investigated nanotechnology-based formulations to improve ophthalmic delivery of ASX. Specifically, nanosized liposomes enable efficient encapsulation of highly lipophilic molecules, promoting prolonged residence time, controlled release and enhanced delivery to ocular tissues, including posterior segments. In vitro dry eye models have demonstrated that ASX-loaded liposomes significantly reduce cellular apoptosis and ROS production while increasing local drug concentration and bioavailability. Overall, nano formulations appear more effective than conventional systems in overcoming ocular barriers; however, further research is needed to develop delivery systems capable of further improving the stability, solubility and bioavailability of ASX in ophthalmic applications [117].

Regarding the nasal mucosa, ASX has been reported to promote wound healing following endoscopic sinus surgery, often complicated by scarring [120]. However, clinical studies in humans evaluating topical ASX administration to the nasal mucosa remain limited, similar to what is observed for the pharyngeal and vaginal mucosa. Consequently, further preclinical and clinical investigations are required to clarify the efficacy, safety and therapeutic potential of ASX in these areas.

### 4.3. Parenteral Administration

Parenteral administration involves delivering a drug intravenously, intramuscularly, intraperitoneally, or subcutaneously [121,122]. This route bypasses the gastrointestinal tract and first-pass metabolism, allowing the drug to reach the bloodstream or specific tissues and enabling rapid distribution to organs throughout the body, including the brain for an immediate therapeutic effect. It is particularly advantageous for emergency treatments, such as the management of acute pain or life-threatening infections, where rapid action is essential [122]. The parenteral route is indicated when there is a risk of dosing errors, poor adherence to therapy, gastrointestinal intolerance or inadequate response to oral doses [123]. Furthermore, according to Sharma et al. (2024) parenteral administration can improve drug bioavailability compared with oral administration [124]. Although it is invasive and requires qualified healthcare personnel, it remains a preferred choice to overcome absorption barriers and ensure rapid therapeutic action [125]. Regarding ASX, its poor solubility, low stability against light, oxygen and heat, and limited bioavailability make parenteral administration particularly challenging. Resolving these issues is essential for developing ASX-based therapies. For poorly soluble substances like ASX, the parenteral route often requires high concentrations of organic solvents and/or surfactants, increasing the risk of vehicle-related safety issues and potentially altering the molecule’s chemical properties. Parenteral administration is also one of the most promising strategies for delivering drugs to the brain, for example in the treatment of Alzheimer’s disease, but the main obstacle is crossing the blood–brain barrier [126]. To overcome this limitation, ASX-loaded “stealth” lipid nanoparticles (SLNs) were developed using the solvent diffusion technique [127]. However, systemic use of SLNs is limited by the opsonization process, one of the mechanisms used by the immune system to fight pathogens, which is responsible for their short half-life (3–5 min). To prolong circulation time, one common strategy is to reduce or prevent opsonization. One study showed that coating ASX-SLN with the surfactant polysorbate 80 (ASX-SSLN) was most effective. These particles have an average size <200 nm, suitable for parenteral administration, and remain stable for up to six months of storage (long-term stability). During storage, samples were maintained at room temperature and protected from light exposure. ASX-SSLNs proved to be more stable than unloaded SSLNs, probably due to ASX’s antioxidant capacity to preserve the system against instability phenomena [128]. More recently, a glucosylceramide-based nanoemulsion, loaded with ASX, curcumin, and sesame oil, was designed to exploit glucose transporter-mediated uptake to increase the delivery of the encapsulated compounds. The developed formulation achieved a 1.6-fold increase in neuronal uptake and a stronger ROS suppression than control. Moreover, the degradation of ASX was lower than 10% after 30 days of storage at 4 °C and in the dark, showing physical stability due to the protective oil phase and the co-encapsulation with curcumin [129].

Several animal studies have shown that parenterally administered ASX improves insulin resistance and reduces glucose intolerance and hyperglycemia, suggesting a potential preventive role in the development and progression of diabetes [130,131]. Research has also been evaluated synthetic ASX disodium disuccinate (CardASXTM), injected intravenously, as a myocardial protective agent in experimental animal models of infarction (rats, rabbits and dogs) [132]. Intravenous pretreatment with CardASXTM in Sprague Dawley rats resulted in a significant reduction in myocardial infarct size, with markedly lower doses required to achieve comparable effects relative to oral administration [133]. These results indicate that parenteral CardASXTM may have clinical utility for the pretreatment of patients at risk of myocardial infarction [134]. In summary, these studies suggest that parenteral formulations of astaxanthin, particularly injectable derivatives such as CardASXTM, may offer clinically relevant advantages for acute cardioprotection.

### 4.4. Other Routes of Administration

Rectal administration, thanks to the relatively stable rectal environment and low enzymatic activity, is indicated for APIs with poor oral absorption, unstable in the gastric environment or intended to act locally [135]. There is no scientific evidence in the literature describing the use of ASX by the rectal route as the primary route of administration.

In recent years, several studies have suggested a potential protective role of ASX against lung diseases, including lung cancer, but currently clinical trials involving direct pulmonary administration of ASX via aerosols, inhalers or other forms of inhalation are limited [136]. In this context, a recent study by Zhang et al. (2025) explored this approach by developing an inhalation nano-delivery system to prevent radiation-induced lung injury and tested it in murine models [137]. ASX-loaded poly(lactic-co-glycolic acid) (PLGA) nanoparticles modified with chitosan were developed. This nano formulation improved the solubility and permeability of ASX, resulting in significant benefits, including the prevention of ROS production and the inhibition of radiological and pathological changes associated with the development of pulmonary fibrosis.

## 5. Commercial Formulations and Regulatory Aspects

As discussed in previous sections, ASX is widely studied and used in various drug delivery contexts, but most of the currently commercially available formulations are dietary supplements, often in the form of capsules, softgels or tablets. Table 2 shows some examples of commercial ASX products, resulting from the search made on pharmacy retail websites. Among the various formulations available on the market, oral softgels predominate, generally containing ASX in amounts ranging from 4 to 12 mg. In most cases, ASX is the primary ingredient, while in other products it is included as a secondary component in combination with other antioxidants or bioactive molecules with potential health benefits. In contrast, skin topical formulations have been developed mainly as cosmetic products, highlighting the lack of pharmaceutical delivery systems. Therefore, the ASX concentration in these products is not always declared. At the experimental level, several studies have explored topical formulations, such as eye drops. However, these products are poorly available commercially, further highlighting the lack of topical pharmaceutical formulations of ASX on the market. In both cases, the main benefit attributed to ASX is related to its antioxidant activity and its ability to counteract oxidative stress. However, while oral administration enables systemic absorption of the molecule, thereby supporting immune system, skin, eye and cardiovascular health, topical application acts primarily locally, particularly in the anti-aging field (Table 2).

ASX was initially considered a novel food ingredient under Regulation (EC) No. 258/97, which was subsequently repealed and replaced by Regulation (EU) 2015/2283 [138], which introduced the Union list of novel foods, establishing that only novel foods authorized and included in that list, which also includes ASX, may be marketed. Subsequently, Commission Implementing Regulation (EU) 2017/2470 established the Union list of novel foods, including ASX-rich oleoresin derived from the microalgae *Haematococcus pluvialis* [139]. The specifications of this molecule were later updated following a request submitted in 2022 by Astareal AB, aimed at modifying the limits relating to the portion of ASX monoesters and diesters and the fraction of the 9-cis-astaxanthin stereoisomer. These changes were approved based on the favorable opinion of the European Food Safety Authority (EFSA). For human use, Commission Implementing Regulation (EU) 2021/1377 established use in food supplements for adults and adolescents over 14 years of age, with a maximum intake level of 8.0 mg/day of ASX [140]. Subsequently, Commission Implementing Regulation (EU) 2023/1581 extended the use of ASX in food supplements intended for children aged 3 to under 10 years (up to 2.3 mg/day) and adolescents aged 10 to under 14 years (up to 5.7 mg/day) [141]. In the United States, dietary supplements are not subject to prior approval by the Food and Drug Administration (FDA), and some ingredients, such as natural extracts derived from *H. pluvialis* containing ASX esters, are considered Generally Recognized As Safe (GRAS) for specific conditions of use [142]. Specifically, in the GRAS notification submitted by INNOBIO Limited (GRN 580), the mean dietary exposure to ASX was estimated at 0.96 mg/person/day, whereas high-end exposure reaches 1.62 mg/person/day at the 90th percentile among the most frequent users [143].

## 6. Conclusions

ASX is a bioactive compound of large interest for human health due to its remarkable antioxidant properties and associated benefits. However, its low aqueous solubility, chemical instability, sensitivity to light, oxygen and heat, and limited bioavailability make the selection of an appropriate formulation and route of administration extremely important for the maintenance or enhancement of its therapeutic and functional potential. Oral administration is the most common route, especially in the nutraceutical and pharmaceutical sectors; however, gastrointestinal degradation and lipid-based absorption significantly suppress ASX bioavailability. To overcome these limitations, innovative encapsulation systems have been developed to obtain an enhancement of stability, bioavailability and solubility while preserving its antioxidant activity. Topical administration enables localized therapeutic effects, particularly dermatological and ophthalmic applications, whereas parenteral administration emphasizes the importance of formulation to ensure optimal systemic delivery into the organism. Differently from the oral route, for which several commercial products are available on the market such as capsules, tablets and softgels, the topical route of administration remains underexplored, as evidenced by the limited number of commercially available formulations, especially for skin application. Therefore, future research should focus on overcoming the limitations of ASX for the different routes of administration and on developing new formulations to exploit its potential in pharmaceutical, nutraceutical, and cosmetic applications.

## Figures and Tables

**Table 1:** Comparison between hard capsules, softgels and tablets as solid dosage forms for ASX.

	Hard Capsules	Softgels	Tablets
**Dosage form** **structure**	Rigid shell filled with powder, granules or pellets	Elastic gelatin shell containing liquid or semi-solid fills	Compressed solid unit
**Typical ASX** **Physical state**	Solid (e.g., powder, granules)	Dissolved or dispersed in liquid matrices	Solid (often pre-formulated)
**Oral** **bioavailability**	Variable as a function of capsule content (ASX powder, granules or microencapsulated)	Higher than for hard capsules thanks to lipid matrix	Formulation-dependent
**Stability of ASX**	Dependent on humidity and capsule brittleness	Dependent on lipid composition, ideal for oily/lipophilic APIs	Critical without encapsulation
**Oral** **bioavailability**	Variable as a function of capsule content (ASX powder, granules or microencapsulated)	Higher than for hard capsules thanks to lipid matrix	Formulation-dependent

**Table 2:** Commercial astaxanthin-based products orally or topically administered, for different health applications. The commercial products were searched on pharmacy retail websites including Redcare.

Product Name	Dosage Form	ASX form and Dose	Company Name	Application	Administration
AstaReal^®^	Softgels	ASX	Fuji chemical industry	Health supplement	Oral
Astaxanthin	Softgel	4–12 mg ASX	Now Foods	Systemic antioxidant support	Oral
Astaxanthin	Softgel	5 mg-ASX	Solgar global manufacture	Skin health	Oral
Astaxanthin	Softgel	4 mg-ASX	Swanson Health Products	Ocular and skin health	Oral
Astaxcare	Softgel	4 mg-ASX	Life Science Srls	Systemic oxidative stress protection	Oral
Bioaromatase	Tablets	5 mg-ASX (secondary ingredient)	Biogroup S.r.l.	Antioxidant	Oral
Vegavero^®^ Astaxanthin	Vegan capsules	8 mg-ASX	Vanatari International GmbH	Skin, ocular and immune system support	Oral
Astaxanthin Gold^TM^	Softgel	4 mg-ASX	NutriGold	Skin, immune, and eye health	Oral
BioAstin^®^	Softgel Capsules	12 mg-ASX 4 mg-ASX	Nutrex Hawaii /Cyanotech Co.	Ocular, skin, cardiovascular and immune system support	Oral
Guam	Cream Serum	ASX	Lacote Srl	Skin protection and anti-aging effect	Topical
Astaxanthin treatment cream	Cream	ASX	AJ skin care	Anti-aging and antioxidant	Topical
DermaE	Cream	ASX	Topics Pharmaceuticals	Anti-aging and antioxidant	Topical
Hyasta eye drops	Eye drops	Phytocomplex (ASX esters > 50%)	Aveflor a.s.	Ocular health	Topical

## Data Availability

No new data were created or analyzed in this study. Data sharing is not applicable to this article.

## References

[1] Nair A., Ahirwar A., Singh S., Lodhi R., Lodhi A., Rai A., Jadhav D.A., Harish, Varjani S., Singh G. (2023). Astaxanthin as a King of Ketocarotenoids: Structure, Synthesis, Accumulation, Bioavailability and Antioxidant Properties. Mar. Drugs.

[2] Stachowiak B., Szulc P. (2021). Astaxanthin for the Food Industry. Molecules.

[3] Brotosudarmo T.H.P., Limantara L., Setiyono E., Heriyanto (2020). Structures of Astaxanthin and Their Consequences for Therapeutic Application. Int. J. Food Sci..

[4] Seabra L.M.J., Pedrosa L.F.C. (2010). Astaxanthin: Structural and Functional Aspects. Rev. Nutr..

[5] Dutta S., Kumar S.P.J., Banerjee R. (2023). A Comprehensive Review on Astaxanthin Sources, Structure, Biochemistry and Applications in the Cosmetic Industry. Algal Res..

[6] Maoka T. (2020). Carotenoids as Natural Functional Pigments. J. Nat. Med..

[7] Patil A.D., Kasabe P.J., Dandge P.B. (2022). Pharmaceutical and Nutraceutical Potential of Natural Bioactive Pigment: Astaxanthin. Nat. Prod. Bioprospect..

[8] Kumar S., Kumar R., Kumari A., Panwar A. (2022). Astaxanthin: A Super Antioxidant from Microalgae and Its Therapeutic Potential. J. Basic Microbiol..

[9] Guerin M., Huntley M.E., Olaizola M. (2003). Haematococcus Astaxanthin: Applications for Human Health and Nutrition. Trends Biotechnol..

[10] Sun L., Li Y., Yang A., Xie M., Xiong R., Huang C. (2025). Astaxanthin: A Comprehensive Review of Synthesis, Biological Activities and Applications. Food Chem..

[11] Peng L., Zhang Z., Li Q., Yang H. (2025). Current Challenges and Issues in the Application of Astaxanthin. Fishes.

[12] Zhao T., Yan X., Sun L., Yang T., Hu X., He Z., Liu F., Liu X. (2019). Research Progress on Extraction, Biological Activities and Delivery Systems of Natural Astaxanthin. Trends Food Sci. Technol..

[13] Khayyal M.T., Teaima M.H., Marzouk H.M., El-Hazek R.M., Behnam F., Behnam D. (2024). Comparative Pharmacokinetic Study of Standard Astaxanthin and Its Micellar Formulation in Healthy Male Volunteers. Eur. J. Drug Metab. Pharmacokinet..

[14] Chen Y., Su W., Tie S., Zhang L., Tan M. (2022). Advances of Astaxanthin-Based Delivery Systems for Precision Nutrition. Trends Food Sci. Technol..

[15] Zhu Y., He L., Qin X., Xu M., Tan C. (2025). Recent Advances in Nanodelivery Systems for Astaxanthin: A Review. Food Biosci..

[16] Sun J., Wei Z., Xue C. (2023). Recent Research Advances in Astaxanthin Delivery Systems: Fabrication Technologies, Comparisons and Applications. Crit. Rev. Food Sci. Nutr..

[17] Chen S., Wang J., Feng J., Xuan R. (2023). Research Progress of Astaxanthin Nano-Based Drug Delivery System: Applications, Prospects and Challenges?. Front. Pharmacol..

[18] Jia L., Wang W., Zhao H., Ding X., Zheng M., Cai D., Wang Y., Wang Z., Liu H. (2025). Innovative Nano Delivery Systems for Astaxanthin: Enhancing Stability, Bioavailability, and Targeted Therapeutic Applications. J. Agric. Food Chem..

[19] Kim S.Y., Cho E.A., Yoo J.M., In M.J., Chae H.J. (2008). Solubility and Storage Stability of Astaxanthin. Korean J. Biotechnol. Bioeng..

[20] Jannel S., Caro Y., Bermudes M., Petit T. (2020). Novel Insights into the Biotechnological Production of *Haematococcus pluvialis*-Derived Astaxanthin: Advances and Key Challenges to Allow Its Industrial Use as Novel Food Ingredient. J. Mar. Sci. Eng..

[21] Higuera-Ciapara I., Félix-Valenzuela L., Goycoolea F.M. (2006). Astaxanthin: A Review of Its Chemistry and Applications. Crit. Rev. Food Sci. Nutr..

[22] Koller M., Muhr A., Braunegg G. (2014). Microalgae as Versatile Cellular Factories for Valued Products. Algal Res..

[23] Ravi S., Ambati R.R., Kamath S.B., Chandrappa D., Narayanan A., Chauhan V.S., Ravishankar G.A. (2012). Influence of Different Culture Conditions on Yield of Biomass and Value Added Products in Microalgae. Dyn. Biochem. Process Biotechnol. Mol. Biol..

[24] Zhang B.Y., Geng Y.H., Li Z.K., Hu H.J., Li Y.G. (2009). Production of Astaxanthin from Haematococcus in Open Pond by Two-Stage Growth One-Step Process. Aquaculture.

[25] Mussagy C.U., Caicedo-Paz A.V., Farias F.O., Tropea A., La Tella R., Guzmán-Flores J.M., Mondello L., Herculano R.D., Filho P.E.L.L., Piazza R.D. (2025). Comparative Analysis of Bacterial and Microalgal Natural Astaxanthin: Part I—Focus on Composition, Molecular Interactions, Antioxidant Activities, Physicochemical and Biological Functions. Algal Res..

[26] Bhatt P.C., Ahmad M., Panda B.P. (2013). Enhanced Bioaccumulation of Astaxanthin in *Phaffia rhodozyma* by Utilising Low-Cost Agro Products as Fermentation Substrate. Biocatal. Agric. Biotechnol..

[27] Mussagy C.U., Khan S., Kot A.M. (2022). Current Developments on the Application of Microbial Carotenoids as an Alternative to Synthetic Pigments. Crit. Rev. Food Sci. Nutr..

[28] Nishida Y., Berg P., Shakersain B., Hecht K., Takikawa A., Tao R., Kakuta Y., Uragami C., Hashimoto H., Misawa N. (2023). Astaxanthin: Past, Present, and Future. Mar. Drugs.

[29] Yin C., Yang S., Liu X., Yan H. (2013). Efficient Extraction of Astaxanthin from *Phaffia rhodozyma* with Polar and Non-Polar Solvents after Acid Washing. Chin. J. Chem. Eng..

[30] Carré P. (2021). About Solvents Used in the Preparation of Oils for Cosmetic Products Complying with the Cosmos Standard. OCL.

[31] Sarada R., Vidhyavathi R., Usha D., Ravishankar G.A. (2006). An Efficient Method for Extraction of Astaxanthin from Green Alga *Haematococcus pluvialis*. J. Agric. Food Chem..

[32] Ni H., Chen Q., He G., Wu G., Yang Y. (2008). Optimization of Acidic Extraction of Astaxanthin from *Phaffia rhodozyma*. J. Zhejiang Univ. Sci. B.

[33] Kim B., Youn Lee S., Lakshmi Narasimhan A., Kim S., Oh Y.-K. (2022). Cell Disruption and Astaxanthin Extraction from *Haematococcus pluvialis*: Recent Advances. Bioresour. Technol..

[34] Quitério E., Grosso C., Ferraz R., Delerue-Matos C., Soares C. (2022). A Critical Comparison of the Advanced Extraction Techniques Applied to Obtain Health-Promoting Compounds from Seaweeds. Mar. Drugs.

[35] Butler T., Golan Y. (2020). Astaxanthin Production from Microalgae. Microalgae Biotechnology for Food, Health and High Value Products.

[36] Harith Z.T., de Andrade Lima M., Charalampopoulos D., Chatzifragkou A. (2020). Optimised Production and Extraction of Astaxanthin from the Yeast Xanthophyllomyces Dendrorhous. Microorganisms.

[37] Khoo K.S., Ooi C.W., Chew K.W., Foo S.C., Lim J.W., Tao Y., Jiang N., Ho S.-H., Show P.L. (2021). Permeabilization of *Haematococcus pluvialis* and Solid-Liquid Extraction of Astaxanthin by CO_2_-Based Alkyl Carbamate Ionic Liquids. Chem. Eng. J..

[38] Moon G., Lee N., Kang S., Park J., Kim Y.-E., Lee S.-A., Chitumalla R.K., Jang J., Choe Y., Oh Y.-K. (2021). Hydrothermal Synthesis of Novel Two-Dimensional α-Quartz Nanoplates and Their Applications in Energy-Saving, High-Efficiency, Microalgal Biorefineries. Chem. Eng. J..

[39] Lee N., Narasimhan A.L., Moon G., Kim Y.-E., Park M., Kim B., Mahadi R., Chung S., Oh Y.-K. (2022). Room-Temperature Cell Disruption and Astaxanthin Recovery from *Haematococcus Lacustris* Cysts Using Ultrathin α-Quartz Nanoplates and Ionic Liquids. Appl. Sci..

[40] Ausich R.L. (1997). Commercial Opportunities for Carotenoid Production by Biotechnology. Pure Appl. Chem..

[41] (2024). The European Commision implementing regulation. (EU) 2024/1026 Amending Implementing Regulation (EU) 2017/2470 as Regards the Specifications of the Novel Food Astaxanthin-Rich Oleoresin from Haematococcus pluvialis Algae.

[42] Malcangi G., Inchingolo A.M., Casamassima L., Trilli I., Ferrante L., Longo M., Inchingolo F., Marinelli G., Palermo A., Dipalma G. (2026). The Role of Astaxanthin as an Antioxidant and Anti-Inflammatory Agent in Human Health: A Systematic Review. Int. J. Mol. Sci..

[43] Davinelli S., Nielsen M.E., Scapagnini G. (2018). Astaxanthin in Skin Health, Repair, and Disease: A Comprehensive Review. Nutrients.

[44] Tso M.O., Lam T.T. (1996). Method of Retarding and Ameliorating Central Nervous System and Eye Damage. U.S. Patent.

[45] Donoso A., González-Durán J., Muñoz A.A., González P.A., Agurto-Muñoz C. (2021). Therapeutic Uses of Natural Astaxanthin: An Evidence-Based Review Focused on Human Clinical Trials. Pharmacol. Res..

[46] Zhang X.-S., Zhang X., Wu Q., Li W., Wang C.-X., Xie G.-B., Zhou X.-M., Shi J.-X., Zhou M.-L. (2014). Astaxanthin Offers Neuroprotection and Reduces Neuroinflammation in Experimental Subarachnoid Hemorrhage. J. Surg. Res..

[47] Arefpour H., Rasaei N., Amini M.R., Salavatizadeh M., Hashemi M., Makhtoomi M., Hajiaqaei M., Gholizadeh M., Askarpour M., Hekmatdoost A. (2024). The Effects of Astaxanthin Supplementation on Liver Enzyme Levels. Int. J. Vitam. Nutr. Res..

[48] Chen Y.-Y., Lee P.-C., Wu Y.-L., Liu L.-Y. (2015). In Vivo Effects of Free Form Astaxanthin Powder on Anti-Oxidation and Lipid Metabolism with High-Cholesterol Diet. PLoS ONE.

[49] Nagaraj S., Rajaram M.G., Arulmurugan P., Baskaraboopathy A., Karuppasamy K., Jayappriyan K.R., Sundararaj R., Rengasamy R. (2012). Antiproliferative Potential of Astaxanthin-Rich Alga *Haematococcus pluvialis* Flotow on Human Hepatic Cancer (HepG2) Cell Line. Biomed. Prev. Nutr..

[50] Tominaga K., Hongo N., Karato M., Yamashita E. (2012). Cosmetic Benefits of Astaxanthin on Humans Subjects. Acta Biochim. Pol..

[51] Vitale M., Gomez-Estaca J., Chung J., Chua S.-C., Pampanin D.M. (2025). Encapsulation Techniques to Enhance Astaxanthin Utilization as Functional Feed Ingredient. Mar. Drugs.

[52] Zhu Y., He L., Xu M., Qin X., Tan C. (2025). A Review on Emerging Carriers to Improve Bioaccessibility and Bioavailability of Astaxanthin. J. Future Foods.

[53] Panagiotakopoulos I., Nasopoulou C. (2024). Extraction Methods, Encapsulation Techniques, and Health Benefits of Astaxanthin. Sustainability.

[54] Li B., Lee J.-Y., Luo Y. (2023). Health Benefits of Astaxanthin and Its Encapsulation for Improving Bioavailability: A Review. J. Agric. Food Res..

[55] Oninku B., Lomas M.W., Burr G., Aryee A.N.A. (2025). Astaxanthin: An Overview of Its Sources, Extraction Methods, Encapsulation Techniques, Characterization, and Bioavailability. J. Agric. Food Res..

[56] Mayersohn M. (1987). Drug Absorption. J. Clin. Pharmacol..

[57] Kumar S., Gill B.S., Verma A., Verma M.L., Kushwaha R. (2020). Biotechnological Production of High-Valued Algal Astaxanthin and Lutein under Different Growth Conditions. Biotechnological Production of Bioactive Compounds.

[58] Fransico N. (2023). The Role of Routes of Administration in Drug Absorption and Bioavailability. J. Pharmacol. Rep..

[59] Mastropietro D., Park K., Omidian H. (2017). 4.23 Polymers in Oral Drug Delivery. Comprehensive Biomaterials II.

[60] Murakami T. (2017). Absorption Sites of Orally Administered Drugs in the Small Intestine. Expert Opin. Drug Discov..

[61] Chitchumroonchokchai C., Failla M.L. (2017). Bioaccessibility and Intestinal Cell Uptake of Astaxanthin from Salmon and Commercial Supplements. Food Res. Int..

[62] Ito M., Ghosh A., Nishida Y., Honda M. (2026). Plasma Appearance and Tissue Distribution of Astaxanthin Isomers in Male Sprague–Dawley Rats after Oral Administration of *Z*-isomer-enriched Astaxanthin Esters through Thermal Treatment. J. Sci. Food Agric..

[63] Abdol Wahab N.R., Meor Mohd Affandi M.M.R., Fakurazi S., Alias E., Hassan H. (2022). Nanocarrier System: State-of-the-Art in Oral Delivery of Astaxanthin. Antioxidants.

[64] Jiang W., Badehnoosh B., Ruan W. (2025). Food-Grade Nanostructured Delivery Systems for Oral Administration of Astaxanthin: Bioprocessing Strategies and Therapeutic Applications. npj Sci. Food.

[65] Gao C., Gong N., Chen F., Hu S., Zhou Q., Gao X. (2024). The Effects of Astaxanthin on Metabolic Syndrome: A Comprehensive Review. Mar. Drugs.

[66] Vertzoni M., Augustijns P., Grimm M., Koziolek M., Lemmens G., Parrott N., Pentafragka C., Reppas C., Rubbens J., Van Den Abeele J. (2019). Impact of Regional Differences along the Gastrointestinal Tract of Healthy Adults on Oral Drug Absorption: An UNGAP Review. Eur. J. Pharm. Sci..

[67] Mercke Odeberg J., Lignell Å., Pettersson A., Höglund P. (2003). Oral Bioavailability of the Antioxidant Astaxanthin in Humans Is Enhanced by Incorporation of Lipid Based Formulations. Eur. J. Pharm. Sci..

[68] Saini R.K., Prasad P., Lokesh V., Shang X., Shin J., Keum Y.-S., Lee J.-H. (2022). Carotenoids: Dietary Sources, Extraction, Encapsulation, Bioavailability, and Health Benefits—A Review of Recent Advancements. Antioxidants.

[69] Ito N., Saito H., Seki S., Ueda F., Asada T. (2018). Effects of Composite Supplement Containing Astaxanthin and Sesamin on Cognitive Functions in People with Mild Cognitive Impairment: A Randomized, Double-Blind, Placebo-Controlled Trial. J. Alzheimer’s Dis..

[70] Gullapalli R.P., Mazzitelli C.L. (2017). Gelatin and Non-Gelatin Capsule Dosage Forms. J. Pharm. Sci..

[71] Wuytens P., Parakhonskiy B., Yashchenok A., Winterhalter M., Skirtach A. (2014). Pharmacological Aspects of Release from Microcapsules—From Polymeric Multilayers to Lipid Membranes. Curr. Opin. Pharmacol..

[72] Palomero-Hernández F.J., Caballo-González M.Á., de la Mata F.J., García-Gallego S. (2025). Sustainable Shell Formulations as Alternative to the Conventional Soft Gelatin Capsules in Pharmaceutical and Nutraceutical Applications. A Review. Macromol. Mater. Eng..

[73] Phelan D., Prado-Cabrero A., Nolan J. (2017). Stability of Commercially Available Macular Carotenoid Supplements in Oil and Powder Formulations. Nutrients.

[74] Choi H.D., Kang H.E., Yang S.H., Lee M.G., Shin W.G. (2011). Pharmacokinetics and First-Pass Metabolism of Astaxanthin in Rats. Br. J. Nutr..

[75] Ruiz-Núñez B., E Schuitemaker G., Dijck-Brouwer D.J., AJ Muskiet F. (2014). Kinetics of Plasma and Erythrocyte-Astaxanthin in Healthy Subjects Following a Single and Maintenance Oral Dose. J. Young Pharm..

[76] Kanakaraj L. (2024). Preparation and Evaluation of Soft Gelatin Capsules Loaded with Astaxanthin, Niacin and Garlic Oil for Removal of Plague in Atherosclerosis. J. Med. Pharm. Allied Sci..

[77] Martínez-Álvarez Ó., Calvo M.M., Gómez-Estaca J. (2020). Recent Advances in Astaxanthin Micro/Nanoencapsulation to Improve Its Stability and Functionality as a Food Ingredient. Mar. Drugs.

[78] Zhao Y., Liu J., Zhang S., Wang Z., Jia H., Oda H., Li R. (2022). Fabrication and Characterization of the H/J-Type Aggregates Astaxanthin/Bovine Serum Albumin/Chitosan Nanoparticles. Int. J. Biol. Macromol..

[79] Zhu Y., Gu Z., Liao Y., Li S., Xue Y., Firempong M.A., Xu Y., Yu J., Smyth H.D., Xu X. (2022). Improved Intestinal Absorption and Oral Bioavailability of Astaxanthin Using Poly (Ethylene Glycol)-graft-chitosan Nanoparticles: Preparation, in Vitro Evaluation, and Pharmacokinetics in Rats. J. Sci. Food Agric..

[80] Liao Y., Wang H., Li S., Xue Y., Chen Y., Adu-Frimpong M., Xu Y., Yu J., Xu X., Smyth H.D.C. (2024). Preparation of Astaxanthin-loaded Composite Micelles with Coaxial Electrospray Technology for Enhanced Oral Bioavailability and Improved Antioxidation Capability. J. Sci. Food Agric..

[81] Sorasitthiyanukarn F.N., Muangnoi C., Rojsitthisak P., Rojsitthisak P. (2022). Chitosan Oligosaccharide/Alginate Nanoparticles as an Effective Carrier for Astaxanthin with Improving Stability, in Vitro Oral Bioaccessibility, and Bioavailability. Food Hydrocoll..

[82] Zhang W., Zhang X., Lv X., Qu A., Liang W., Wang L., Zhao P., Wu Z. (2024). Oral Delivery of Astaxanthin via Carboxymethyl Chitosan-Modified Nanoparticles for Ulcerative Colitis Treatment. Molecules.

[83] Solymosi K., Latruffe N., Morant-Manceau A., Schoefs B. (2015). Food Colour Additives of Natural Origin. Colour Additives for Foods and Beverages.

[84] Umamaheswari D., Abdul Hasan Shathali A., Umarani G., Sheik Abdulla Kapoor M., Vinodha G., Balaji R., Ponraj S. (2024). Self-Micro Emulsifying Drug Delivery System (SMEDDS): An Innovative Tool To Improve Bioavailability. Int. J. Res. Pharmacol. Pharmacother. (IJRPP).

[85] Li Y., Wei Q., Su J., Zhang H., Fan Z., Ding Z., Wen M., Liu M., Zhao Y. (2024). Encapsulation of Astaxanthin in OSA-Starch Based Amorphous Solid Dispersions with HPMCAS-HF/Soluplus^®^ as Effective Recrystallization Inhibitor. Int. J. Biol. Macromol..

[86] Huang J., Feng X., Zhang S., Wang L., Yue J., Chu L. (2023). Preparation and Characterization of Astaxanthin-loaded Microcapsules and Its Application in Effervescent Tablets. J. Sci. Food Agric..

[87] Aung W.T., Khine H.E.E., Chaotham C., Boonkanokwong V. (2022). Production, Physicochemical Investigations, Antioxidant Effect, and Cellular Uptake in Caco-2 Cells of the Supersaturable Astaxanthin Self-Microemulsifying Tablets. Eur. J. Pharm. Sci..

[88] Sangnim T., Huanbutta K. (2020). Development and Evaluation of Astaxanthin Orally Disintegrating Tablets Prepared from Coprocessed Excipients for Use in the Elderly. Key Eng. Mater..

[89] Rivera-Hernández G., Roether J.A., Aquino C., Boccaccini A.R., Sánchez M.L. (2025). Delivery Systems for Astaxanthin: A Review on Approaches for in Situ Dosage in the Treatment of Inflammation Associated Diseases. Int. J. Pharm..

[90] Luo F., Wang S., Zhang X., Liu Z., Zhu R., Xue W. (2024). Extraction of Astaxanthin from *Haematococcus pluvialis* and Preparation of Astaxanthin Liposomes. Molecules.

[91] Liu C., Zhang S., McClements D.J., Wang D., Xu Y. (2019). Design of Astaxanthin-Loaded Core–Shell Nanoparticles Consisting of Chitosan Oligosaccharides and Poly(Lactic-*Co*-Glycolic Acid): Enhancement of Water Solubility, Stability, and Bioavailability. J. Agric. Food Chem..

[92] Liu C., Liu Z., Sun X., Zhang S., Wang S., Feng F., Wang D., Xu Y. (2018). Fabrication and Characterization of β-Lactoglobulin-Based Nanocomplexes Composed of Chitosan Oligosaccharides as Vehicles for Delivery of Astaxanthin. J. Agric. Food Chem..

[93] Li M., Zahi M.R., Yuan Q., Tian F., Liang H. (2016). Preparation and Stability of Astaxanthin Solid Lipid Nanoparticles Based on Stearic Acid. Eur. J. Lipid Sci. Technol..

[94] Favas R., Almeida H., Peixoto A.F., Ferreira D., Silva A.C., Favas R., Almeida H., Peixoto A.F., Ferreira D., Silva A.C. (2024). Advances in Encapsulating Marine Bioactive Compounds Using Nanostructured Lipid Carriers (NLCs) and Solid Lipid Nanoparticles (SLNs) for Health Applications. Pharmaceutics.

[95] Shehata M.K., Ismail A.A., Kamel M.A. (2023). Ombined Donepezil with Astaxanthin via Nanostructured Lipid Carriers Effective Delivery to Brain for Alzheimer’s Disease in Rat Model. Int. J. Nanomed..

[96] Makhmalzade B., Chavoshy F. (2018). Polymeric Micelles as Cutaneous Drug Delivery System in Normal Skin and Dermatological Disorders. J. Adv. Pharm. Technol. Res..

[97] Miller T.R. (1992). Principles of Therapeutics. Vet. Clin. N. Am. Equine Pract..

[98] Wang S., Wei Y., Wang Y., Cheng Y. (2023). Cyclodextrin Regulated Natural Polysaccharide Hydrogels for Biomedical Applications-a Review. Carbohydr. Polym..

[99] Janagam D.R., Wu L., Lowe T.L. (2017). Nanoparticles for Drug Delivery to the Anterior Segment of the Eye. Adv. Drug Deliv. Rev..

[100] Lima S.G.M., Freire M.C.L.C., Oliveira V.D.S., Solisio C., Converti A., De Lima Á.A.N. (2021). Astaxanthin Delivery Systems for Skin Application: A Review. Mar. Drugs.

[101] Geng Q., Zhao Y., Wang L., Xu L., Chen X., Han J. (2020). Development and Evaluation of Astaxanthin as Nanostructure Lipid Carriers in Topical Delivery. AAPS PharmSciTech.

[102] Ponto T., Latter G., Luna G., Leite-Silva V.R., Wright A., Benson H.A.E. (2021). Novel Self-Nano-Emulsifying Drug Delivery Systems Containing Astaxanthin for Topical Skin Delivery. Pharmaceutics.

[103] Kaur R., Arora V., Goswami M. (2024). Exploring Astaxanthin-Loaded Nanoformulations for Skin Targeting: Present Progress and Prospective Directions. Curr. Drug Ther..

[104] Binsi P.K., Parvathy U., Jeyakumari A., George Thomas N., Zynudheen A.A. (2025). Marine Biopolymers in Cosmetics. Marine Biopolymers.

[105] Bin-Jumah M., Alwakeel S.S., Moga M., Buvnariu L., Bigiu N., Zia-Ul-Haq M. (2021). Application of Carotenoids in Cosmetics. Carotenoids: Structure and Function in the Human Body.

[106] Sun C., Liu Z., Li Y., Wang J., Jiang Y., Zhao C. (2025). Beyond Antioxidant Monotony: Astaxanthin’s Multi-Axis Anti-Ageing Mechanisms, and Innovations in Delivery Technology-A Review. J. Future Foods.

[107] Lee Y.S., Jeon S.H., Ham H.J., Lee H.P., Song M.J., Hong J.T. (2020). Improved Anti-Inflammatory Effects of Liposomal Astaxanthin on a Phthalic Anhydride-Induced Atopic Dermatitis Model. Front. Immunol..

[108] Oh H., Lee J.S., Sung D., Lim J.-M., Choi W. (2020). Potential Antioxidant and Wound Healing Effect of Nano-Liposol with High Loading Amount of Astaxanthin. Int. J. Nanomed..

[109] Barari F., Maghsoudian S., Alinezhad V., Ahmadi S.M., Amiri F.T., Fatahi Y., Barari M., Ebrahimnejad P., Akbari J., Atyabi F. (2025). Effective Topical Delivery of Astaxanthin via Optimized Chitosan-Coated Nanostructured Lipid Carriers: A Promising Strategy for Enhanced Wound Healing and Tissue Regeneration. J. Drug Deliv. Sci. Technol..

[110] Ekofitranto R., Zulaikhah S.T., Wibowo J.W. (2025). In Vivo Study on the Effect of Astaxanthin Cream on Preventing Inflammation in Skin Tissue Exposed to Acute UVB by Reducing MDA and IL-6 Levels. MEDISAINS J. Ilm. Ilmu-Ilmu Kesehat..

[111] Zakaria N.N.A., Zamzurie N.A., Harith Z.T. (2021). Evaluation of Sunscreen Cream Incorporated with Astaxanthin from *Haematococcus pluvialis* in Different Storage Conditions. IOP Conf. Ser. Earth Environ. Sci..

[112] Tosato M.G., Orallo D.E., Fangio M.F., Diz V., Dicelio L.E., Churio M.S. (2016). Nanomaterials and Natural Products for UV-Photoprotection. Surface Chemistry of Nanobiomaterials.

[113] Aishwarya Baarathi G., Bose V.B.S.C., Veerichetty V. (2023). Anti Tyrosinase Activity of Astaxanthin Cream Formulation for Hyper Pigmentation. Int. J. Pharm. Res. Appl..

[114] Nurdianti LSumarli R.I.S., Setiawan F., Gustaman F. (2022). Pengembangan sediaan blush on cream astaxanthin sebagai pewarna alami. J. Pharmacopolium.

[115] Cheng X.-Y., Xiong Y.-J., Yang M.-M., Zhu M.-J. (2019). Preparation of Astaxanthin Mask from *Phaffia rhodozyma* and Its Evaluation. Process Biochem..

[116] Giannaccare G., Pellegrini M., Senni C., Bernabei F., Scorcia V., Cicero A.F.G. (2020). Clinical Applications of Astaxanthin in the Treatment of Ocular Diseases: Emerging Insights. Mar. Drugs.

[117] Jafari Z., Bigham A., Sadeghi S., Dehdashti S.M., Rabiee N., Abedivash A., Bagherzadeh M., Nasseri B., Karimi-Maleh H., Sharifi E. (2022). Nanotechnology-Abetted Astaxanthin Formulations in Multimodel Therapeutic and Biomedical Applications. J. Med. Chem..

[118] Ren F., Rao C., Xiang Q., Wen J., Dai Q., Li H., Liang J., Chen Y., Peng C. (2025). Production Methods, Biological Activity and Potential Application Prospects of Astaxanthin. Foods.

[119] Luviano M., Zúñiga-González O.G., López-Naranjo E.J., Navarro-Partida J., Orozco-Guareño E., Ramírez-Arreola D.E., González-Ortiz L.J. (2025). Efficient Liposomal Encapsulation and Intraocular Diffusion of Astaxanthin in a Topical Ophthalmic Delivery System. Mater. Lett..

[120] Manciula L.-G., Berce C., Tabaran F., Trombitaș V., Albu S. (2019). The Effects of Postoperative Astaxanthin Administration on Nasal Mucosa Wound Healing. J. Clin. Med..

[121] Wieruszewski J. (2000). Astaxanthin Bioavailabilky, Retention Efficiency and Kinetics in Atlantic Salmon (*SaIrno salar*) as Lnfluenced by Pigment Concentration and Method of Administration (Kinetics Only). Doctoral Dissertation.

[122] Chavhan R. (2025). Nanosuspensions: Enhancing Drug Bioavailability through Nanonization. Ann. Pharm. Fr..

[123] Carrascosa J.M., de la Cueva P., Ara M., Puig L., Bordas X., Carretero G., Ferrándiz L., Sánchez-Carazo J.L., Daudén E., López-Estebaranz J.L. (2016). Methotrexate in Moderate to Severe Psoriasis: Review of the Literature and Expert Recommendations. Actas Dermo-Sifiliogr. (Engl. Ed.).

[124] Sharma R., Kumar S., Malviya R., Prajapati B.G., Puri D., Limmatvapirat S., Sriamornsak P. (2024). Recent Advances in Biopolymer-Based Mucoadhesive Drug Delivery Systems for Oral Application. J. Drug Deliv. Sci. Technol..

[125] Ahmed T.A., El-Say K.M., Ahmed O.A.A., Zidan A.S. (2018). Sterile Dosage Forms Loaded Nanosystems for Parenteral, Nasal, Pulmonary and Ocular Administration. Nanoscale Fabrication, Optimization, Scale-Up and Biological Aspects of Pharmaceutical Nanotechnology.

[126] Ilić T., Đoković J.B., Nikolić I., Mitrović J.R., Pantelić I., Savić S.D., Savić M.M. (2023). Parenteral Lipid-Based Nanoparticles for CNS Disorders: Integrating Various Facets of Preclinical Evaluation towards More Effective Clinical Translation. Pharmaceutics.

[127] Abdelazim K., Ghit A., Assal D., Dorra N., Noby N., Khattab S.N., El Feky S.E., Hussein A. (2023). Production and Therapeutic Use of Astaxanthin in the Nanotechnology Era. Pharmacol. Rep..

[128] Santonocito D., Raciti G., Campisi A., Sposito G., Panico A., Siciliano E., Sarpietro M., Damiani E., Puglia C. (2021). Astaxanthin-Loaded Stealth Lipid Nanoparticles (AST-SSLN) as Potential Carriers for the Treatment of Alzheimer’s Disease: Formulation Development and Optimization. Nanomaterials.

[129] Poyenyate K., Yata T., Angkanaporn K. (2026). In Vitro Investigation of Glucosylceramide-Mediated Brain-Targeting in Water-in-Oil-in-Water Nanoemulsions Containing Astaxanthin, Curcumin, and Sesame Oil. ACS Omega.

[130] Landon R., Gueguen V., Petite H., Letourneur D., Pavon-Djavid G., Anagnostou F. (2020). Impact of Astaxanthin on Diabetes Pathogenesis and Chronic Complications. Mar. Drugs.

[131] Rad N.R., Movahedian A., Feizi A., Aminorroaya A., Aarabi M.H. (2022). Antioxidant Effects of Astaxanthin and Metformin Combined Therapy in Type 2 Diabetes Mellitus Patients. Res. Pharm. Sci..

[132] Lockwood S., Jackson H., Gross G. (2006). Retrometabolic Syntheses of Astaxanthin (3,3-Dihydroxy-β, β-Carotene-4,4-Dione) Conjugates: A Novel Approach to Oral and Parenteral Cardioprotection. Cardiovasc. Hematol. Agents Med. Chem..

[133] Gross G.J., Hazen S.L., Lockwood S.F. (2006). Seven Day Oral Supplementation with Cardax^TM^ (Disodium Disuccinate Astaxanthin) Provides Significant Cardioprotection and Reduces Oxidative Stress in Rats. Mol. Cell. Biochem..

[134] Gross G.J., Lockwood S.F. (2004). Cardioprotection and Myocardial Salvage by a Disodium Disuccinate Astaxanthin Derivative (Cardax^TM^). Life Sci..

[135] Rathi R., Sanshita, Kumar A., Vishvakarma V., Huanbutta K., Singh I., Sangnim T. (2022). Advancements in Rectal Drug Delivery Systems: Clinical Trials, and Patents Perspective. Pharmaceutics.

[136] Cheng J., Eroglu A. (2021). The Promising Effects of Astaxanthin on Lung Diseases. Adv. Nutr..

[137] Zhang D., He J., Cui J., Wang R., Zhou M. (2025). Inhalable Nano-Astaxanthin for Radiation-Induced Lung Injury via Enhanced Lung Retention and Inflammation Suppression. ACS Appl. Mater. Interfaces.

[138] (2015). Regulation (EU) 2015/2283 of the European Parliament and of the Council of 25 November 2015 on Novel Foods, Amending Regulation (EU) No 1169/2011 of the European Parliament and of the Council and Repealing Regulation (EC) No 258/97 of the European Parliament and of the Council and Commission Regulation (EC) No 1852/2001.

[139] (2017). Commission Implementing Regulation (EU) 2017/2470 of 20 December 2017 Establishing the Union List of Novel Foods in Accordance with Regulation (EU) 2015/2283 of the European Parliament and of the Council on Novel Foods.

[140] (2021). Commission Implementing Regulation (EU) 2021/1377 of 19 August 2021 Authorising the Change of the Conditions of Use of the Novel Food Astaxanthin-Rich Oleoresin from Haematococcus pluvialis Algae Under Regulation (EU) 2015/2283 of the European Parliament and of the Council and Amending Commission Implementing Regulation (EU) 2017/2470.

[141] (2023). Commission Implementing Regulation (EU) 2023/1581 of 1 August 2023 Amending Implementing Regulation (EU) 2017/2470 as Regards the Conditions of Use of the Novel Food ‘Astaxanthin-Rich Oleoresin from Haematococcus pluvialis Algae’.

[142] U.S. Food and Drug Administration (2015).

[143] U.S. Food and Drug Administration (2017).

